# Co-fermented yellow wine lees by *Bacillus subtilis* and *Enterococcus faecium* regulates growth performance and gut microbiota in finishing pigs

**DOI:** 10.3389/fmicb.2022.1003498

**Published:** 2022-10-21

**Authors:** Yu Zhang, Cheng Wang, Weifa Su, Zipeng Jiang, Huan He, Tao Gong, Lixia Kai, Huangen Xu, Yizhen Wang, Zeqing Lu

**Affiliations:** ^1^National Engineering Research Center for Green Feed and Healthy Breeding, Zhejiang University, Hangzhou, Zhejiang, China; ^2^Key Laboratory of Molecular Nutrition, Ministry of Education, Zhejiang University, Hangzhou, Zhejiang, China; ^3^Key Laboratory of Animal Nutrition and Feed, Ministry of Agriculture, Zhejiang University, Hangzhou, Zhejiang, China; ^4^Key Laboratory of Animal Nutrition and Feed Science of Zhejiang Province, Institute of Feed Science, Zhejiang University, Hangzhou, Zhejiang, China

**Keywords:** fermented feed, finishing pigs, growth performance, gut microbiota, meat quality, immune status

## Abstract

Fermented yellow wine lees (FYWL) are widely used to increase feed utilization and improve pig performance. Based on the preparation of co-FYWL using *Bacillus subtilis* and *Enterococcus faecalis*, the purpose of this study was to investigate the effects of co-FYWL on growth performance, gut microbiota, meat quality, and immune status of finishing pigs. 75 pigs were randomized to 3 treatments (5 replicates/treatment), basal diet (Control), a basal diet supplemented with 4%FYWL, and a basal diet supplemented with 8%FYWL, for 50 days each. Results showed that the 8% FYWL group significantly reduced the F/G and increased the average daily weight gain of pigs compared to the control group. In addition, 8% FYWL improved the richness of *Lactobacillus* and *B. subtilis* in the gut, which correlated with growth performance, serum immune parameters, and meat quality. Furthermore, acetate and butyrate in the feces were improved in the FYWL group. Simultaneously, FYWL improved the volatile flavor substances of meat, increased the content of flavor amino acids, and played a positive role in the palatability of meat. In addition, FYWL increased serum IgA, IgM, IL-4 and IL-10 levels. Overall, the growth performance, the gut microbiota associated with fiber degradation, meat quality, and immune status were improved in the 8% FYWL group.

## Introduction

There is a shortage of feed resources in China, but the output of yellow distiller grains is enormous (Hu et al., 2014; Yao et al., 2021). Due to improper using, resources are wasted (Li et al., 2013). Yellow wine lees are rich in crude protein (Yao et al., 2018). The residual soluble dietary fiber in the production of yellow rice wine can be used as an energy source for livestock and poultry (Wang et al., 2010), but its amino acid composition is unbalanced. Therefore, in recent years, researchers have adopted probiotic fermentation to improve the nutritional status of the feed. Studies have shown that feeding probiotic fermented diets can improve growth performance, feed conversion, nutritional digestion and gut health of finishing pigs (Thu et al., 2011).

Yellow wine lees are rich in dietary fiber (Choi et al., 2013). The prebiotics that are most often researched include dietary fiber (Gamage et al., 2018). The hindgut microbiota frequently ferment dietary fiber to produce microbial metabolites, including volatile fatty acids (VFA) (Tremaroli and Bäckhed, 2012; Slavin, 2013; Janssen and Kersten, 2015). *Lactobacillus* can improve blood biochemistry and antioxidant status as well as absorption of nutrients (Dowarah et al., 2018). Animals given *Lactobacillu*s FT28 have been shown to have greater villi height and crypt depth, indicating better nutritional use by the organism (Joysowal et al., 2018). Probiotics produce lactic acid, which forms an acidic environment that inhibits the growth of mold in fermented feed (FF) and prevents nutrient loss from secondary fermentation (Yang et al., 2006). *Bacillus subtilis* dietary supplementation enhances antioxidant capacity, immunological status, and growth performance, boosts SCFA synthesis, and regulates the gut microbiota (Ruiz Sella et al., 2021; Xu et al., 2021).

Therefore, in this experiment, yellow wine lees, one of the new alternative feed materials, and bran were used as fermentation substrates to prepare mixed feed through co-fermentation with *B. subtilis* and *Enterococcus faecalis*. The aim of this study is to investigate the effects of new mixed fermented diets on growth performance, meat quality, immune status and gut microbiota of finishing pigs.

## Materials and methods

### Fermented mixed feed preparation

*B. subtilis* ZJU12 (China General Microbiological Culture Collection Center NO:12852) was isolated from pickled vegetables. *Enterococcus faecium* was isolated from the laboratory of Zhejiang University. FYWL was carried out in Kesheng Feed Co., Ltd., Zhejiang, PR of China. 80% of yellow wine lees, and 20% of bran compose up a basic substrate, ending up with 35% per moisture content in sterile water. *B. subtilis* ZJU12 (3 × 10^8^ cfu/g) and *E. faecium* (1 × 10^8^ cfu/g) were added to the mixed substrate and fermented in a FF bag with a one-way exhaust valve at 30°C for 72 h. The nutritional composition analysis of the UFYWL and FYWL is shown in Table 1.

**Table 1:** The chemical analysis of the UFYWL and FYWL.

Item	UFYWL	FYWL
Crude protein, %	24.39 ± 0.62^b^	29.43 ± 0.53^a^
TCA-SP, %	4.14 ± 0.42^b^	7.32 ± 0.39^a^
Small peptides, %	2.11 ± 0.45^b^	3.79 ± 0.47^a^
Reducing sugar, %	2.81 ± 0.15^b^	3.23 ± 0.23^a^
*CF*, %	5.28 ± 0.55	5.17 ± 0.45
ADF, %	4.77 ± 0.55^a^	4.46 ± 0.48^b^
NDF, %	12.24 ± 1.13^a^	12.03 ± 1.35^b^
Total P, %	0.57 ± 0.03	0.64 ± 0.05
pH	4.02 ± 0.01	4.01 ± 0.01
*Enterococcus faecalis*, cfu/g	-	2.9 × 10^8^
*Bacillus subtilis*, cfu/g	-	2.2 × 10^7^

Analyzed values determined in duplicate. Means followed with different superscript letters (a, b) within each line are significantly different (*p*< 0.05).

### Experimental animals and experimental design

All steps were approved by the Institutional Animal Care and Use Committee at Zhejiang University. Seventy-five fattened pigs (Duroc × Long White × Large White) with an average initial weight of 89.59 ± 1.30 kg were randomly divided into three treatment groups. Each treatment consisted of five replicates per group, with five pigs per replicate. The three treatments were the control group on the basal diet and the experimental group with 4% or 8% FYWL. Six pigs were selected from each treatment after 50 days slaughtered to determine carcass and meat quality. The diets in the experiment followed NRC (2012). The composition and nutritional value of the diets are shown in Table 2. Feed and water were provided *ad libitum* throughout the experiment.

**Table 2:** The composition and nutritional content of the diet.

Item	Control	4%FYWL	8%FYWL
Ingredients, %			
Corn	47.50	44.10	40.60
Barley	15	15	15
Peeled soybean meal	10	9	6
Rice bran	8	8	8
Rice bran meal	8	8	8
Wheat	5	5	5
Fermented yellow wine lees	-	4	8
Premix	4	4	4
Soya oil	2.50	2.90	2.90
Puffed soybean	-	-	2.50
Total, %	100	100	100
Nutrition composition			
GE, MJ/kg	14.11	14.12	14.21
CP, %	12.22	12.41	12.45
*CF*, %	4.52	4.28	3.96
Ca, %	0.79	0.79	0.80
Total P, %	0.73	0.73	0.73
Phe, %	0.52	0.51	0.55
Lys, %	1.49	1.49	1.50
Leu, %	0.81	1.02	1.06
Thr, %	0.54	0.53	0.53
Pro, %	0.65	0.65	0.68
Ala, %	0.72	0.85	0.89

Provided quantities of the following vitamins per kilogram of the complete diet: 6480 I.U. (international units) Vitamin A, 2800 I.U. Vitamin D_3_, 26 mg Vitamin E, 2 mg Vitamin K_3_, 50 mg Vitamin B_1_, 4 mg Vitamin B_2_, 3.0 mg Vitamin B_6_, 0.03 mg Vitamin B_12_, 23 mg D-Pantothenic acid, 20 mg niacin, 1.2 mg Folic acid, 0.2 mg Biotin, 300 mg Choline chloride, 95 mg copper sulfate, 200 mg Ferrous Sulfate, 0.35 mg Potassium iodide, 30 mg Manganese Sulfate, 0.3 mg Sodium selenite, 100 mg Zinc sulfate.

### Growth performance

Feed was recorded daily and pigs were weighed on the first and last day of the experiment. Average daily feed intake (ADFI), average daily gain (ADG) and feed/gain ratio (F/G) were then calculated.

### Sample collection

Six pigs in each group were randomly selected and fasting for 16 hours before slaughter on the same day, and blood was collected. Then the serum was separated and stored at −80°C. Samples of the longest dorsal muscle (LM) were collected to determine pork quality, amino acids, Inosine-5′-monophosphate, alcohol, aldehyde, and ketone. Fresh feces were collected daily for 5 days before slaughter, part of which was stored at −20°C to determine the apparent digestibility of nutrients, and part of which was stored at −80°C for determining SCFA.

### Chemical analysis

According to the AOAC International recommendations, the fodder was evaluated for CP, ether extract, NDF, ADF, Ca, and total P. The endogenous indicator method was used to determine the contents of crude protein, crude fat, calcium, phosphorus and crude fiber in feces (Verschuren et al., 2021). The feces used to measure SCFA were filtered and determined by capillary GC after filtering (GC-2010 plus; Shimadzu, Kyoto, Japan) (Yang et al., 2020; Zhao et al., 2022). Observe the intestinal morphology with hematoxylin and eosin staining (Cao et al., 2019) by transmission electron microscopy.

Inosine-5′-monophosphate was measured according to the method of Lee et al. (2017). The determination of alcohol, aldehyde, and ketone was carried out with HS-SPME-GC–MS (Li et al., 2022). And the pretreatment and determination of amino acids were performed according to the method of “Determination of amino acids in food” (GB/T 5009.124–2003). pH values of meat were measured using a pH meter. a*, b*, and L* of samples from the different pigs were measured using a colorimeter. To determine drip loss, meat was measured by using EZ-DripLoss method (Huang et al., 2020).

The levels of serum IL-4, IL-6, and IL-10 were measured according to the instructions using the pig source assay kit (Jiangsu Meibiao Biotechnology Co., Ltd., Jiangsu P.R. China). SOD, GSH-Px, CAT, MDA, and T-AOC were detected by using commercial assay kits (Nanjing Jiancheng Bioengineering Institute, Nanjing, China) according to the instructions.

### Microbial analysis of feces

FastDNA SPIN Kit for Soil (MP Biomedicals Ltd., United States) was used to extract the total genomic DNA from the feces samples. The V3-V4 gene region of the bacterial 16S rRNA gene was amplified with the primers 338F (5’-ACTCCTACGGGAGGCAGCAG-3′) and 806R (5’-GGACTACHVGGGTWTCTAAT-3′). A PCR was performed at 95°C for 3 min, followed by 27 cycles of denaturing at 95°C for 30 s, annealing at 55°C for 30 s and extension at 72°C for 45 s, and single extension at 72°C for 10 min, and 10°C until halted by the user. PCR products were then extracted, purified and quantified. Purified amplicons were collected and sequenced on the Illumina MiSeq PE300 platform (Illumina, San Diego, United States). The raw reads were deposited into the NCBI Sequence Read Archive (SRA) database (Accession Number: PRJNA860351). At last, the data were processed using the QIIME package (V1.7.0).^1^

### Statistical analysis

All data were statistically analyzed using SPSS 19.0 software. One-way ANOVA and Duncan test were used to determine the mean difference. At *p* < 0.05, the difference between treatments was significant. Bar graphs were generated in GraphPad Prism 7.

## Results

### The chemical analysis of the UFYWL and FYWL

Table 1 shows the nutritional content of UFYWL and FYWL. FYWL had the higher content of CP, TCA-SP and P compared to the UFYWL, which were 20.66, 76.81 and 12.28% higher than that of UFYWL, respectively.

### Effects of supplementation with FYWL on the growth performance of finishing pigs

Compared with the control group, there was no significant difference in ADFI and ADG among the groups (*p* > 0.05), but FYWL group tended to increase the average daily gain and daily feed intake, and the 8% FYWL group markedly reduced F/G (*p* < 0.05; Table 3).

**Table 3:** Effects of supplementation with FYWL (4 and 8%) on the growth performance in finishing pigs.

Item	Control	4%FYWL	8%FYWL
Initial body weight, kg	89.27 ± 2.11	89.30 ± 0.38	90.20 ± 1.21
Final body weight, kg	125.28 ± 0.93	126.05 ± 1.3	129.63 ± 2.51
ADFI, kg/d	2.78 ± 0.01	2.81 ± 0.02	2.82 ± 0.01
ADG, kg/d	0.92 ± 0.01	0.93 ± 0.01	0.95 ± 0.02
F/G	3.03 ± 0.02^a^	3.01 ± 0.04^a^	2.98 ± 0.06^b^

The data is the mean of 5 replicates per treatment. Means without a common letter are significantly different (*p* < 0.05).

### Effect of FYWL on digestibility and intestinal morphology of finishing pigs

**Figure 1:**
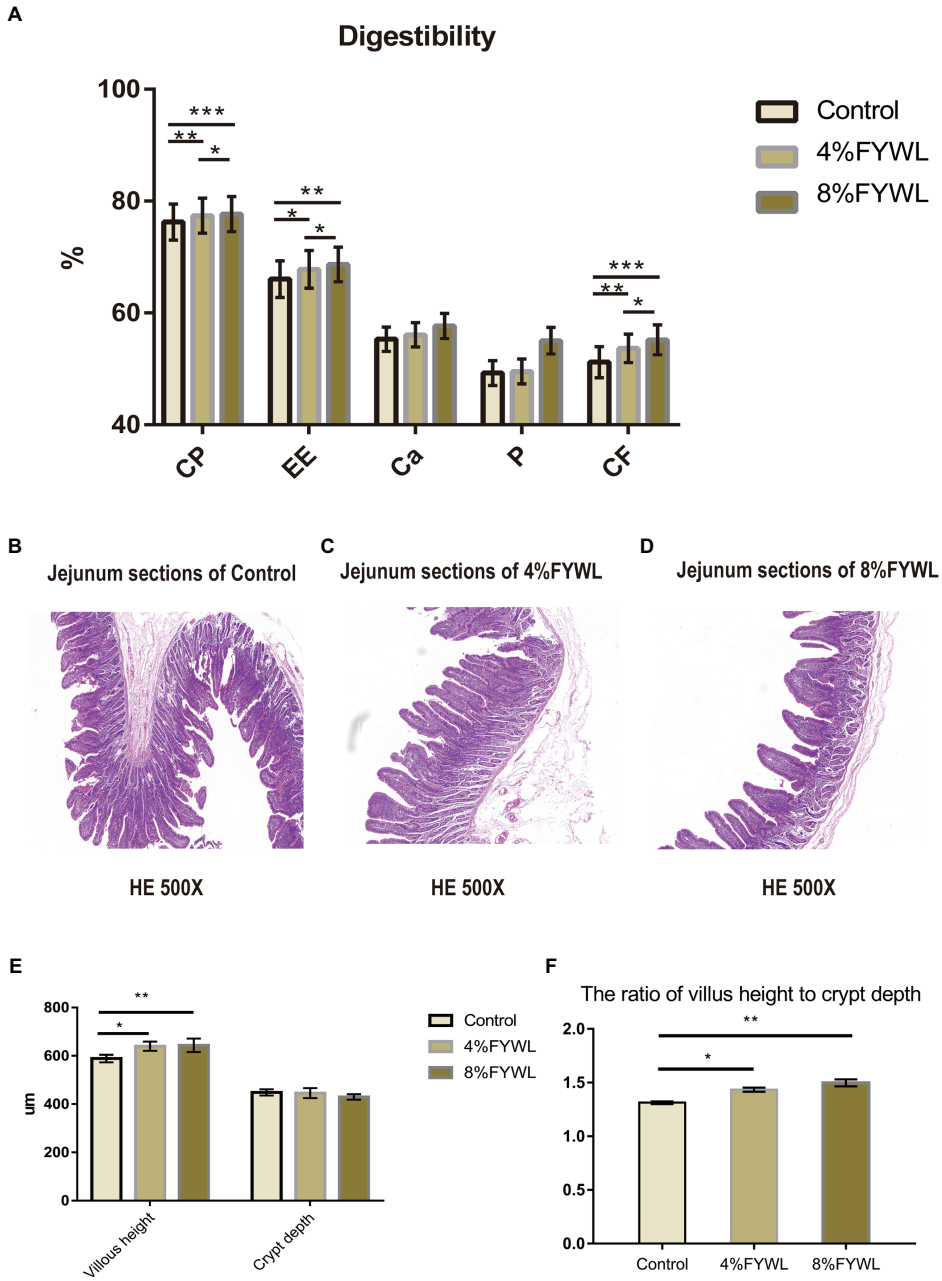
Nutrient digestibility and jejunal morphology. **(A)** Effect of fermented feed on the apparent digestibility of intestinal nutrients in fattening pigs, CP, crude protein; EE, ether extract; *CF*, crude fiber. **(B–D)** Pig intestine stained with H&E. **(E,F)** Effect of fermented feed on the length of intestinal villi and depth of crypt in finishing pigs. *, **, and *** represented significant differences (*p* < 0.05), (*p* < 0.01) and (*p* < 0.001) from indicated control groups, respectively.

As shown in Figure 1A, the addition of FF to the diet was beneficial in improving the nutrient digestibility of pigs, and the FYWL improved the apparent nutrient digestibility of crude protein, crude fat, calcium, phosphorus and crude fiber. Among them, the apparent digestibility of crude protein, crude fat, calcium, and crude fiber was best improved by adding 8% FYWL to the diet compared with the control group. AS shown in Figures 1B–D, we can visually see the villi height and crypt depth. From Figures 1E,F, it can be seen that the FYWL group can significantly improve the villi height and the ratio of villus height to crypt depth, and there was no significant difference between the 4% FYWL and 8% FYWL.

### Effect of FYWL on gut microbiota of finishing pigs

**Figure 2:**
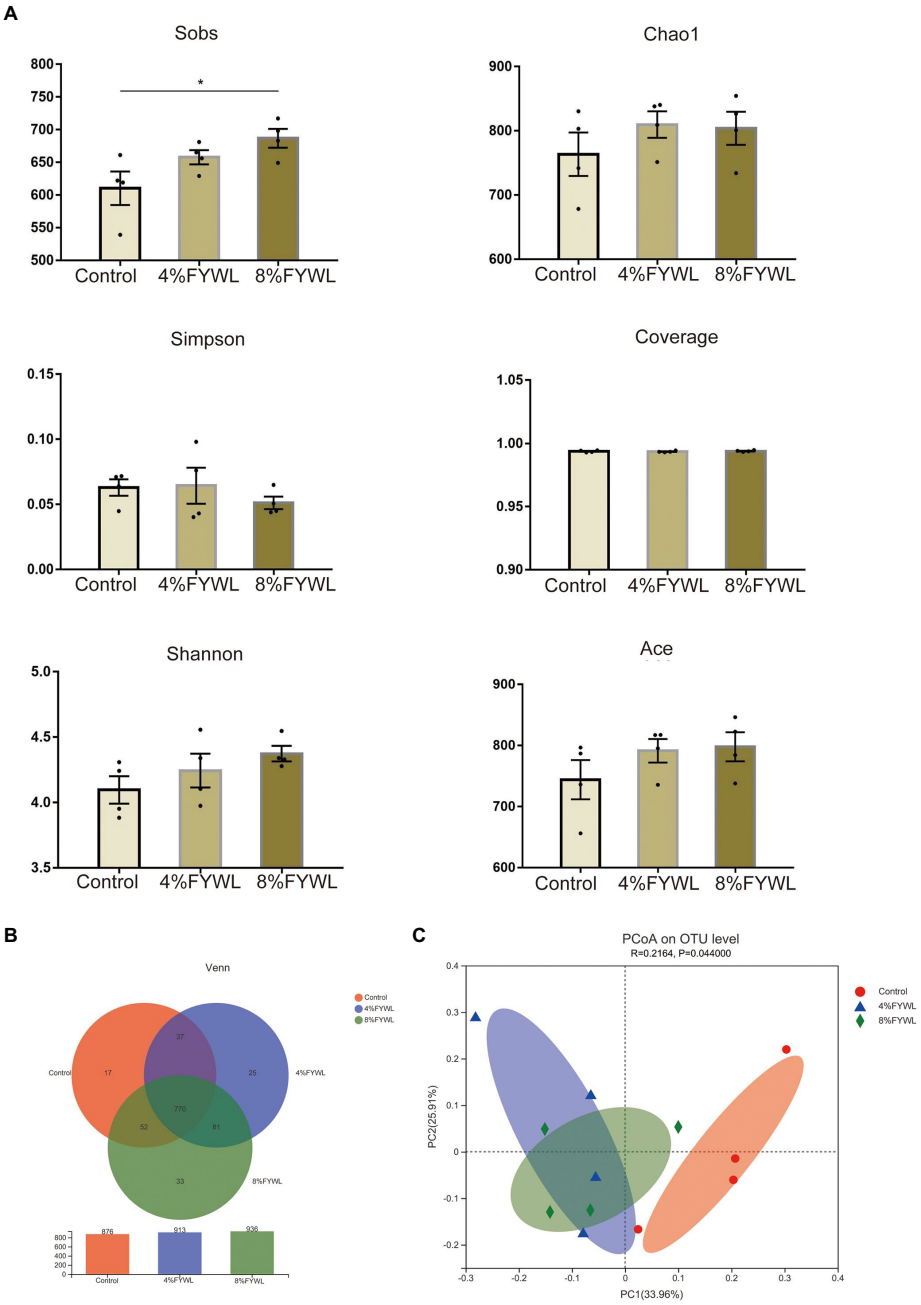
Diversity and composition of microbiota in different groups. **(A)** Column of α diversity indices of bacterial community based on 16S rRNA sequencing. **(B)** Venn diagram. **(C)** Principal co-ordinates (PCoA) analyzes.

Three groups of pig manure were sequenced to achieve Good’s coverage of 0.99 (Figure 2A), suggesting that the sequencing depth is sufficient for microbiological structural analysis. To explore the diversity of the microbial community, we examined α-diversity indices at the species level. The results showed that the control group was significantly less abundant than the 8% FYWL (Wilcoxon rank-sum test *p* < 0.05; Figure 2A). In addition, the FYWL showed a tendency to increase in the Ace index, Chao1 index, and Shannon index. This suggests that FYWL may increase the gut microbial diversity in pigs. Venn (Figure 2B) shows the shared and specific OUTs of different groups. The unique OUT numbers of the control group, the 4%FYWL and the 8% FYWL, were 17, 25 and 33, respectively, and the number of common OUTs of the three groups is 770. To understand differences in microbial composition between the three groups, we calculated Bray-Curtis distances at the species level in each sample group as part of the β-diversity measures. Principal coordinates analysis (PCoA) (Figure 2C) revealed significant differences between the three groups. Analysis of similarity (ANOSIM) analysis also showed that the microbial composition of the different groups was significantly different (*p* < 0.05).

**Figure 3:**
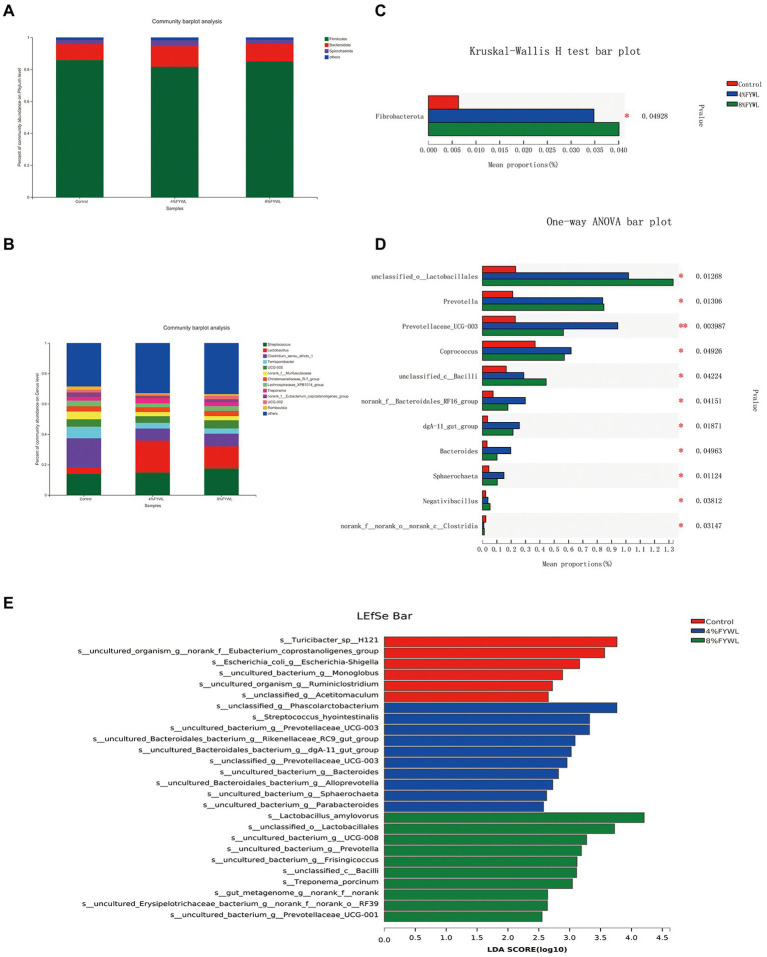
Effect of different feeds on the relative abundance of different bacterial species. **(A)** Phylum-level of the bacterial community in feces. **(B)** Genus-level of the bacterial community in feces. **(C)** Phylum-level in Kruskal–Wallis H. **(D)**, Genus-level in one-way ANOVA. **(E)** Linear discriminant analysis LDA scores (>2.5) were computed for features. Letters represented the taxonomy of the bacteria: p, phylum, c, class; o, order; f, family; g, genus.

At the phylum level and genus level, the microbial composition differed between treatment groups (Figure 3). *Firmicutes*, *Bacteroidota*, and *Spirochaete* are the dominant microflora of fattening pigs. *Clostridium_sensu_stricto_1, Terrisporobacter, norank_f__Muribaculaceae,* and *Lachnospiraceae_XPB1014_group* decreased. Figure 3C shows that the FYWL group increased the abundance of *Fibrobacterota* (*p* < 0.05), Figure 3D showed that *Lactobacillales*, *Prevotella*, *Coprococcus*, *Bacilli*, *dgA-11 gut group*, *Bacteroides*, *Sphaerochaeta*, and *Negativibacillus* (*p* < 0.05) abundances. We performed LDA coupled with effect size (LEfSe) on the taxa with LDA scores greater than 2.5, and the results are shown in Figure 3E. The most influential bacterial group structure in the Control was *Turicibacter*, *Eubacterium_coprostanoligenes,* and *Escherichia-Shigella,* The most influential genera on the 4% FYWL were *Phascolarctobacterium, streptococcus-hyointestinalis,prevotellaceae, Rikenellaceae, dgA-11 gut group, Prevotellaceae UCG 003, Bacteroides, Alloprevotella, Sphaerochaeta, Parabacteroides.* The most influenced by the 8% FYWL group was *Lactobacillus_amylovorus, Lactobacillales, Prevotella, Frisingicoccus, Bacilli, Treponema_porcinum.*

### Effect of FYWL on faecal SCFAs of finishing pigs

The content of short-chain fatty acids (SCFAs) in the feces is shown in Table 4. 8% FYWL group had significantly higher acetic acid and butyric acid content than the Control (*p* < 0.05). Meanwhile, propionate, isobutyrate, valerate, and isovalerate have an increasing trend in the FYWL group.

**Table 4:** Effect of FYWL on content of SCFA.

Item	Control	4%FYWL	8%FYWL
Acetate, mg/g	2.56 ± 0.43^b^	2.90 ± 0.28^b^	3.48 ± 0.23^a^
Propionate, mg/g	1.43 ± 0.31	1.53 ± 0.17	1.69 ± 0.26
Isobutyrate, mg/g	0.15 ± 0.04	0.17 ± 0.04	0.20 ± 0.03
Butyrate, mg/g	0.83 ± 0.17^b^	0.91 ± 0.13^ab^	1.09 ± 0.07^a^
Valerate, mg/g	0.28 ± 0.03	0.29 ± 0.04	0.31 ± 0.03
Isovalerate, mg/g	0.17 ± 0.07	0.21 ± 0.01	0.22 ± 0.05

The data is the mean of 6 replicates per treatment. Means without a common letter are significantly different (*p* < 0.05).

### Effects of FYWL on the meat quality of finishing pigs

The 8% FYWL group significantly improved the marbling score, meat color score, a* (*p* < 0.05). There was also a facilitation effect on the loin eye muscle area. FYWL had a significant increase in intramuscular fat (*p* < 0.05) and inosine-5′-monophosphate content (*p* < 0.05) compared to the control group (Table 5).

**Table 5:** Effect of FYWL on meat quality of finishing pig.

Item	Control	4%FYWL	8%FYWL
Live weight, kg	131.08 ± 3.38	131.00 ± 2.55	131.17 ± 1.86
Carcass weight, kg	95.92 ± 1.72	95.42 ± 2.15	97.25 ± 1.04
Dressing percentage, %	0.73 ± 0.03	0.73 ± 0.01	0.74 ± 0.01
Drip loss,%	3.29 ± 1.85	3.12 ± 1.91	2.42 ± 0.58
Backfat thickness, mm	25.11 ± 4.16	26.82 ± 2.44	29.25 ± 3.42
Loin eye area,cm^2^	63.60 ± 1.29	66.97 ± 5.26	67.90 ± 8.80
Intramuscular fat,%	1.71 ± 0.13^b^	2.10 ± 0.25^a^	2.15 ± 0.08^a^
Marbling score	1.90 ± 0.42^ab^	1.90 ± 0.22^ab^	2.60 ± 0.55^a^
Inosine-5′-monophosphate, mg/g	1.98 ± 0.20^c^	2.05 ± 0.32^b^	2.26 ± 0.50^a^
Meat color score	2.08 ± 0.49^b^	2.58 ± 0.58^ab^	3.08 ± 0.58^a^
L^*^	47.79 ± 3.59	46.73 ± 5.55	46.28 ± 2.16
a^*^	10.48 ± 0.74^b^	12.24 ± 1.09^ab^	13.01 ± 2.36^a^
b^*^	10.09 ± 1.27	9.45 ± 1.04	9.25 ± 0.50
pH	5.48 ± 0.09	5.51 ± 0.11	5.56 ± 0.17

The value is the average of 6 repetitions per treatment. No identical letter indicates significant difference (*p* < 0.05). L^*^ indicates lightness, while a^*^ and b^*^ indicate chromaticity.

The content of amino acids in muscle, especially flavor amino acids, such as Asp., Pro, Glu, Gly, Thr, Ser and Ala, was closely related to the flavor of the meat. As shown in Table 6, 8%FYWL was the most effective for the improvement of flavor and indispensable amino acids in muscle. The changes in volatile flavor substances are shown in Supplementary Material. In comparison, pigs fed FYWL showed an improvement in volatile flavor compounds. Specifically, the addition of 8% FYWL to the feed significantly increased the content of aldehydes such as hexanal, heptanal, acetaldehyde (*p* < 0.05), and significantly increased the content of (E)-3-octen-2-ol, cis-menthyl-2,8-dienol (*p* < 0.05), and 2-pentylfuran (*p* < 0.05). The experimental results showed that 8% FYWL had a better enhancement effect on the enhancement of volatile flavor substances.

**Table 6:** Effect of FYWL on amino acid content of meat.

Item	Control	4%FYWL	8%FYWL
Asp	2.25 ± 0.03^c^	2.36 ± 0.07^b^	2.53 ± 0.06^a^
Ser	0.97 ± 0.06^b^	1.03 ± 0.06^ab^	1.06 ± 0.04^a^
Glu	3.98 ± 0.02^b^	3.98 ± 0.03^b^	4.17 ± 0.04^a^
Gly	1.00 ± 0.03^b^	1.04 ± 0.02^ab^	1.05 ± 0.04^a^
Ala	1.25 ± 0.17^b^	1.39 ± 0.06^ab^	1.47 ± 0.04^a^
Cys	0.39 ± 0.02	0.36 ± 0.04	0.34 ± 0.04
Tyr	0.82 ± 0.01	0.83 ± 0.04	0.86 ± 0.04
Pro	0.90 ± 0.03	0.90 ± 0.02	0.91 ± 0.05
Thr	1.11 ± 0.07^b^	1.18 ± 0.03^ab^	1.21 ± 0.04^a^
Val	0.46 ± 0.04^c^	0.55 ± 0.02^b^	0.60 ± 0.02^a^
Met	1.19 ± 0.05^a^	1.09 ± 0.05^b^	1.06 ± 0.05^b^
Ile	1.18 ± 0.06^a^	1.11 ± 0.06^ab^	1.04 ± 0.04^b^
Leu	1.88 ± 0.06^c^	1.98 ± 0.06^b^	2.11 ± 0.04^a^
Phe	0.92 ± 0.01^b^	0.94 ± 0.05^b^	1.04 ± 0.09^a^
Lys	2.17 ± 0.05^c^	2.29 ± 0.06^b^	2.41 ± 0.08^a^
His	1.08 ± 0.09	1.07 ± 0.05	1.06 ± 0.05
Arg	1.50 ± 0.06	1.54 ± 0.08	1.59 ± 0.08

The data is the mean of 6 replicates per treatment. Means without a common letter are significantly different (*p* < 0.05).

### Effects of FYWL on serum antioxidant ability and immunity of finishing pigs

Compared to the control group, T-AOC, GSH-Px, and CAT activities were significantly higher (*p* < 0.05) in the pigs fed 8% FYWL, and MDA content was significantly lower (*p* < 0.05) in the pigs fed 8% FYWL (Table 7).

**Table 7:** Effects of FYWL on serum antioxidant ability of finishing pigs.

Item	Control	4%FYWL	8%FYWL
T-AOC, U/mL	2.43 ± 0.08^b^	2.54 ± 0.12^ab^	2.64 ± 0.07^a^
GSH-Px, U/mL	813.01 ± 5.63^b^	817.68 ± 6.07^b^	835.39 ± 4.63^a^
CAT, U/mL	5.52 ± 0.14^c^	5.73 ± 0.15^b^	6.43 ± 0.11^a^
SOD, U/mL	60.57 ± 2.03	61.53 ± 1.71	62.23 ± 1.71
MDA, nmol/mL	2.54 ± 0.09^a^	2.52 ± 0.07^ab^	2.41 ± 0.07^b^

The data is the mean of 6 replicates per treatment. Means without a common letter are significantly different (*p* < 0.05).

For serum immunity (Table 8), FYWL significantly increased the level of IL-4. The level of IL-6 was significantly decreased (*p* < 0.05). Serum indicators were measured to assess serum immunoglobulin concentration. Differences in IgG levels between the groups were not significant, but IgA and IgM were significantly increased in 8% FYWL group (*p* < 0.05).

**Table 8:** Effect of FYWL on serum immunity.

Item	Control	4%FYWL	8%FYWL
IL-6, pg./mL	1215.33 ± 69.02^a^	1118.52 ± 69.33^b^	1039.60 ± 46.38^c^
IL-4, pg./mL	94.88 ± 3.69^b^	100.36 ± 3.12^b^	110.50 ± 5.37^a^
IL-10, pg./mL	706.03 ± 14.08	729.62 ± 12.42	732.05 ± 7.38
lgA, g/L	0.59 ± 0.05^c^	0.61 ± 0.07^b^	0.63 ± 0.03^a^
lgM, g/L	0.28 ± 0.01^c^	0.34 ± 0.02^b^	0.36 ± 0.01^a^
lgG, g/L	0.90 ± 0.06	0.93 ± 0.07	0.98 ± 0.07

The data is the mean of 6 replicates per treatment. Means without a common letter are significantly different (*p* < 0.05).

### Spearman’s correlation among gut microbiota, meat quality, serum index, and SCFA

**Figure 4:**
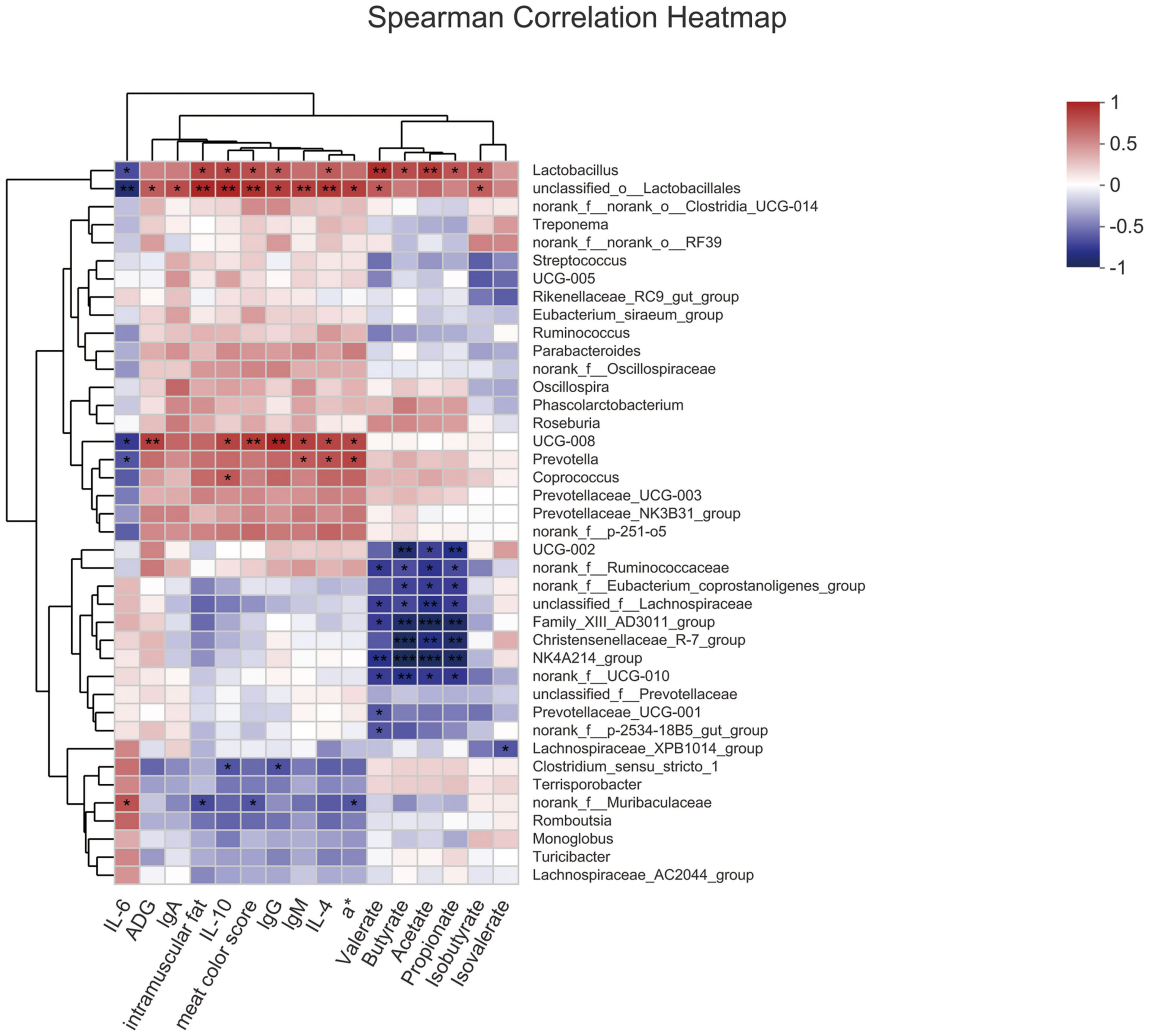
Spearman’s correlation among gut microbiota, serum index, meat quality and SCFAs. * and ** represented significant differences (*p* < 0.05) and (*p* < 0.01) from indicated control groups, respectively.

Analysis of Spearman correlation to study the relationship among gut microbiota, meat quality, serum index, and SCFA (Figure 4). *Lactobacillus*, which was enriched in the 8% FYWL group and positively correlated with intramuscular fat, IL-10, meat color score, lgG, IL-4, butyrate, propionate, isobutyrate (*p* < 0.05), was positively related to valerate, acetate (*p* < 0.01) and was a negative correlation with IL-6 (*p* < 0.05). *Prevotella* enriched in FYWL was positively correlated with lgM, IL-4, a* (*p* < 0.05), and negatively with IL-6 (*p* < 0.05). On the contrary, *Ruminococcaceae*, *Lachnospiracea*, and *Eubacterium coprostanoligenes* were abundant in control group. *Ruminococcaceae*, and *Lachnospiraceae* had a passive correlation with valerate, butyrate, acetate, propionate (*p* < 0.05). Interestingly, *Eubacterium coprostanoligenes* were negatively correlated with butyrate, acetate, and propionate (*p* < 0.05).

## Discussion

In this study, yellow wine lees (YWL) co-fermented by *B. subtilis* and *Enterococcus faecalis* could regulate growth performance, intestinal flora, meat quality, and immune status of finishing pigs. Interestingly, the beneficial effect of 8% FYWL was superior to 4% FYWL. YWL is the main by-product of yellow rice wine industry, and carbohydrates can be used as an important energy source for microorganisms. Its amino acid composition is unbalanced, and improper treatment of residual alcohol may cause intestinal diseases in animals. The fermentation of probiotics improved the crude protein and polypeptide content of YWL with high efficiency. The results showed that FYWL contained more proteins and TCA-SP compared to YWL. Crude protein, acid-soluble protein, ANF, and pH are important indicators to assess the nutritional value of feeds. TCA-SP are easily available in the intestine and have antioxidant and immunomodulatory functions (Gilbert et al., 2008). Degradation of macromolecular proteins, including antigenic proteins, leads to an increase in TCA-SP. Carbohydrates are used by probiotics leading to an increase in bacterial protein is the main reason for triggering an increase in crude protein in FFs, *B. subtilis* secretes proteases to degrade macromolecular proteins leading to an increase in TCA-SP. Our results showed that FYWL contained more CP and TCA-SP than UFYWL. In addition, *B. subtilis* can also secrete cellulase, hemicellulase and xylanase to increase soluble dietary fiber in feeds. Overall, the synergistic fermentation of bacteria and enzymes improves protein quality by degrading anti-nutritional factors and fiber, which in turn enhances the nutritional value of feeds. It helps to improve the digestibility of nutrients in animals.

One study noted that due to the enrichment of probiotics on the surface and inside the feed and the secretion of extracellular enzymes or organic acids by probiotics, this change is more favorable for the digestive enzymes to work and improve the nutrient utilization of the animal (Xie et al., 2021). Fiber digestibility in pigs is mainly derived from the hindgut flora, and it has been reported that pigs provided with 19.10% dietary fiber contained higher intestinal microbial richness. At the genus level, compared with the control group, the number and types of several fiber-degrading bacteria related to carbohydrate metabolism and energy metabolism increased (Pu et al., 2020). In addition, fiber induced an increase in fiber-degrading bacteria, promoting VFA metabolism, which may help Suhuai pigs grow. It has been suggested that dietary corn bran may improve the growth performance of pigs by increasing butyrate production. Specifically, the *Lactobacillus* in fermented yellow wine lees promote butyric acid production and increase feed utilization efficiency (Lin et al., 2020; Direkvandi et al., 2021).

The increase in the ratio of villus height to crypt depth indicates that the digestion and absorption of nutrients in the small intestine are enhanced. The results of this study suggest that fermented wine lees can improve intestinal morphology, increase the height of intestinal villi and decrease the depth of the crypt, thus increasing the villi to crypt ratio. This also explains one of the reasons for the increased digestibility in the FYWL group (Hedemann et al., 2003). Bacteria capable of producing SCFAs were found to be beneficial in improving the efficiency of feed utilization. Meanwhile, the contribution of cellulosic bacteria to SCFA is significant and will increase the efficiency of feed utilization by the host.

It was found that the more bacteria capable of producing SCFA, the higher the efficiency of feed utilization (Tan et al., 2017; Yang et al., 2017; Quan et al., 2018). The FF can improve animal health, intestinal morphology and immunity (Sun et al., 2015; Liu et al., 2017; Li et al., 2019; Suda et al., 2021) by affecting intestinal flora, so we also measured intestinal flora to investigate the potential causes affecting the apparent indicators of pigs.

The sobs index showed a significant increase in gut microbial diversity in the 8% FYWL. The increase in microbial diversity contributed to the improvement of the intestinal condition. The low pH and probiotics from FYWL prevented pathogenic infections and improved the gut health (Missotten et al., 2015). The results of the LEFSe analysis showed that Bacteria capable of digesting fibers in the control group included the genus *Ruminiclostridium* (Wang et al., 2021). The abundance of *Ruminococcus* was positively correlated with TNF-α, a common pro-inflammatory factor. Previous studies have shown that enrichment of this family is associated with inflammation of the colonic mucosa (Willing et al., 2010). The 4% FYWL was significantly enriched with *Prevotella* and *Phascolarctobacterium*. *Prevotella* can degrade mucin (Wright et al., 2000; de la Cuesta-Zuluaga et al., 2017), has a growth-promoting effect on the organism, and is capable of producing SCFAs (Li et al., 2020). This bacterium is usually associated with fiber-rich feeds (Wu et al., 2011). *Phascolarctobacterium* was positively associated with acetic acid, propionic acid and total VFA production and it has synergistic effect with fiber degradation. The 8% FYWL was significantly enriched with *Lactobacillus amylovorus*, *Lactobacillales* and *Bacilli*. *L. amylovorus* can effectively utilize dietary fiber and oligosaccharides, and its metabolites help prevent pathogenic bacteria-induced damage to the intestinal barrier (Trevisi et al., 2018). The increased abundance of *Lactobacillus* can effectively regulate the intestinal microbiota and reduce pH. FYWL can effectively prevent pathogenic infections by lowering pH (Missotten et al., 2015). The increase in the abundance of *Lactobacillus* can effectively regulate the immune system. Also, the massive proliferation of beneficial bacteria can competitively adhere to intestinal mucosal cells and reduce the chances of pathogenic bacteria colonization (Valeriano et al., 2017). With the use of probiotic FFs, many studies have demonstrated the promotion effect of *B. subtilis* on animal growth performance (Bader et al., 2012; Liu et al., 2012; Chen and Yu, 2020), and even more, studies have demonstrated the improvement of growth performance under heat stress and immune stress (Musa et al., 2019; Sokale et al., 2019; Abdelqader et al., 2020). In line with this result, our study demonstrated that the addition of *B. subtilis* was effective in increasing ADG, ADFI and decreasing F/G, which was related to *B. subtilis* metabolites such as extracellular digestive enzymes and antifungal proteins (Kim et al., 2004; Sahu et al., 2008). Adding *B. subtilis* to the feed also enhances the immunity (Dong et al., 2020; Guo M. et al., 2020) and improves the metabolic function of the intestine (Xu et al., 2018; Rodrigues et al., 2020). Serum immunoglobulins, especially IgA, IgG, and IgM, usually respond to the immune status of the organism (Balan et al., 2019). In agreement with previous studies (Fazelnia et al., 2021), the levels of IgA and IgM were markedly increased in the experimental group supplemented with *B. subtilis*. This reflects the enhanced immune function of the organism.

Some researchers have indicated that *Lactobacillales* can improve the carcass quality and physicochemical properties, sensory attributes (juiciness and appearance) of pork. Animals given *P. acidilactici* FT28 showed higher total ash and CP percentages in their meat (*P* < 0.05) (Joysowal et al., 2018). Some studies have shown that animals treated with *Bacillus* sp. The meat gets higher protein and amino acid content, which is consistent with our findings (Musa et al., 2019). It has been reported that feeding FF can improve meat color, which is consistent with our experimental results (Guo S. et al., 2020). Muscle fat content is important for evaluating the various sensory qualities of meat, such as tenderness, flavor and juiciness (Hocquette et al., 2010; Van Elswyk and McNeill, 2014). FYWL affects finishing pigs’ meat quality by influencing the muscle’s fat deposition (Yin et al., 2017). A study applied to finishing pigs had similar results to ours. The article effectively improved meat quality through SCFA supplementation and verified that SCFA improves carcass traits and meat quality in pigs by regulating fat metabolism (Zhang et al., 2011; Jiao et al., 2021).

Serum immunoglobulins are important immunoreactive molecules. The results of this study showed that the levels of IgA and IgM were markedly higher in the FYWL group compared to the control group. FYWL with *B. subtilis* was effective in increasing the level of immunoglobulins and decreasing the level of pro-inflammatory factors. This is consistent with the previous results (Xu et al., 2021). In addition, it has been reported that dietary fiber improves the immune performance by regulating the secretion of butyric acid to inhibit the secretion of pro-inflammatory factors and promote the secretion of anti-inflammatory factors. Compared to control, pigs supplemented with FYWL in this study observed increased serum levels of IL-4, and IL-10. The reduction in IL-6 suggests FYWL may reduce inflammatory responses without antibiotics. FYWL improves immunity by SCFAs in the FYWL group, especially acetate and butyrate (Parada Venegas et al., 2019; Luu et al., 2020). Butyrate can act by inhibiting pro-inflammatory cytokines. There is ample evidence that many bacteria present in the intestinal tract of animals are effective in improving feed utilization and enhancing the immune capacity of the organism (Corrigan et al., 2015; Waite and Taylor, 2015; Best et al., 2016; Li et al., 2017). It has been noted that due to the degradation of fiber by intestinal microorganisms to produce butyrate, it can indeed enhance the intestinal barrier through the HIF-1 and AMPK signaling pathways thereby (Peng et al., 2009; Kelly et al., 2015). Oxidative stress is a state of imbalance between antioxidants and free radicals that can generate various reactive oxygen species (ROS) in the body. Excess ROS can damage biological macromolecules such as proteins and nucleic acids and produce large amounts of MDA, eventually leading to tissue damage and disease development. At this time, SOD, GSH-Px, and CAT are produced accordingly in the body to remove redundant ROS and maintain the body’s health (Cao et al., 2021). Some studies have shown that probiotic fermentation could increase the content of small peptides, which are substances with antioxidant properties and can improve antioxidant capacity and free radical scavenging capacity, thus affecting the serum antioxidant capacity of finishing pigs. In conclusion, the fermentation of yellow wine lees can increase immunity and serum antioxidant capacity in finishing pigs.

Spearman’s correlation analysis further showed that *Lactobacillus* was significantly associated with immunity, meat quality, and SCFAs in greater numbers. Specifically, the *Lactobacillus* in fermented yellow wine lees promote butyric acid production (Berni Canani et al., 2016; Lin et al., 2020) and regulate butyric acid metabolism to improve immunity (Hao et al., 2021). It has been shown that the flora can alleviate intestinal inflammation by upregulating IL-22 production through the breakdown of dietary fiber to produce butyrate by activating HIF-1α (Yang et al., 2020). Consistent with this finding, butyrate levels were increased in this experiment, while inflammatory cytokine were downregulated. A growing body of evidence suggests that dietary fiber improves immune performance by regulating butyrate secretion to reduce the secretion of inflammatory cytokine and increase the secretion of anti-inflammatory cytokine (Liu et al., 2021). Several papers have confirmed the benefits of propionate and butyrate on ADFI or ADG in pigs (Lu et al., 2012), which is consistent with our findings. Contrary to our results, oral administration of butyrate reduced feed intake and decreased ADG in mice (Li et al., 2018), and this difference may be due to differences in animal models and health status. Intramuscular fat, meat color score, and a* are common indicators to evaluate carcass traits. In agreement with our results, butyrate supplementation was beneficial in improving body lipid content (Mátis et al., 2019) and carcass traits in fattened pigs (Sun et al., 2020). Spearman correlation analysis suggests that dietary fiber from fermented yellow wine lees may improve immune function, meat quality, and body health of fattened pigs through butyrate metabolism.

In short, the FYWL diet shapes the microbiota associated in the colon. These microorganisms, in turn, have a positive impact on the antioxidant, immune and meat quality of the organism.

## Conclusion

In conclusion, this study demonstrated that co-FFs of *B. subtilis* and *Enterococcus faecalis* improved the growth performance, nutrient digestibility, serum antioxidant capacity, immune status and meat quality of finishing pigs. These beneficial results may be regulated by the phylum *Fibrobacter*, *Lactobacillus* and SCFAs. This provides a theoretical basis for the application of FF on finishing pigs.

## Data availability statement

The datasets presented in this study can be found in online repositories. The names of the repository/repositories and accession number(s) can be found in the article/Supplementary material.

## Ethics statement

The animal study was reviewed and approved by the Institutional Animal Care and Use Committee of Zhejiang University.

## Author contributions

YZ: investigation, data curation, and writing-original draft. CW: research and writing–original draft. WS: visualization and investigation. ZJ: supervision. TG: resources and formal analysis. HH: validation. LK: software. HX: resources. YW: supervision, funding acquisition, methodology, software, and writing—review and editing. ZL: conceptualization, supervision, and writing—review and editing. All authors contributed to the article and approved the submitted version.

## Funding

The design of the study and collection, analysis, and interpretation of data were supported by the fund from Science and Technology Projects of Zhejiang (2022C02043, 2021C02008, and CTZB-2020080127), China Agriculture Research System (CARS-35), and National Center of Technology Innovation for Pigs, China Postdoctoral Science Foundation no. 2022M712796.

## Conflict of interest

The authors declare that the research was conducted in the absence of any commercial or financial relationships that could be construed as a potential conflict of interest.

## Publisher’s note

All claims expressed in this article are solely those of the authors and do not necessarily represent those of their affiliated organizations, or those of the publisher, the editors and the reviewers. Any product that may be evaluated in this article, or claim that may be made by its manufacturer, is not guaranteed or endorsed by the publisher.

## Footnotes

^1^ http://qiime.org/scripts/split_libraries_fastq.html

## Abbreviations

FF: Fermented feed
UFYWL: Unfermented yellow wine lees
FYWL: Fermented yellow wine lees
EE: Ether extract
AA: Amino acid
CP: Crude protein
TCA-SP: Trichloroacetic acid soluble protein
*CF*: Crude fiber
ADF: Acid detergent fiber
NDF: Neutral detergent fiber
AOAC: Association of Official Analytical Chemists
SOD: The activities of superoxide dismutase
GSH-Px: Glutathione peroxidase
CAT: Catalase
MDA: Malonaldehyde
T-AOC: Total antioxidant capacity
