# Supplementary material for: Co-fermented yellow wine lees by Bacillus subtilis and Enterococcus faecium regulates growth performance and gut microbiota in finishing pigs
Source: Front Microbiol. 2022 Oct 21;13:1003498. doi: 10.3389/fmicb.2022.1003498 (PMC9633856; doi:10.3389/fmicb.2022.1003498)
Supplement: Supplementary file 1 [file Table_1.DOCX]

Supplementary material. Effect of FYWL on volatile flavor substances of pork.

| Item | Control | 4%FYWL | 8%FYWL |
| --- | --- | --- | --- |
| Aldehyde, ng/g |  |  |  |
| Hexanal | 6.08±0.17^b^ | 6.37±0.08^a^ | 6.50±0.15^a^ |
| Heptanal | 14.83±0.45^b^ | 15.81±0.45^a^ | 16.25±0.31^a^ |
| Acetaldehyde | 3.94±0.04^c^ | 4.36±0.03^b^ | 4.59±0.08^a^ |
| Pentanal | 6.08±0.15^b^ | 6.37±0.07^a^ | 6.50±0.13^a^ |
| Octyl aldehyde | 28.66±0.46^c^ | 30.38±0.33^b^ | 31.15±0.55^a^ |
| Nonanal | 150.89±4.69^b^ | 168.45±5.70^a^ | 173.16±2.63^a^ |
| phenyl aldehyde | 8.06±0.11^c^ | 10.96±0.35^b^ | 11.96±0.54^a^ |
| Trans -2- sebenal | 5.58±0.06^b^ | 6.58±0.19^a^ | 6.66±0.54^a^ |
| (E, E)-2, 4- nonadiene aldehyde | 6.51±0.14^b^ | 6.81±0.44^ab^ | 7.07±0.12^a^ |
| Dodecyl aldehyde | 28.66±0.42^c^ | 30.38±0.31^b^ | 31.15±0.5^a^ |
| (E)-2- hexenal | 3.31±0.31 | 3.49±0.14 | 3.55±0.07 |
| Trans -4- nonanal | 7.44±0.14 | 7.37±0.06 | 7.4±0.07 |
| Kwai aldehyde | 100.49±0.9 | 105.22±2.5 | 106.56±1.89 |
| Trans -2- nonanal | 10.21±0.43 | 10.03±0.51 | 10.27±0.45 |
| P-isopropylbenzaldehyde | 1.09±0.08 | 1.1±0.06 | 1.15±0.11 |
| 2- heptene aldehyde | 2.5±0.08 | 2.46±0.12 | 2.55±0.14 |
| Alcohol, ng/g |  |  |  |
| (E)-3- octene -2- ol | 90.25±1.08^b^ | 94.19±2.2^a^ | 94.26±2.1^a^ |
| 1- octene -3- ol | 4.06±0.1^b^ | 4.20±0.22^ab^ | 4.29±0.09^a^ |
| 1- pentanol | 5.29±0.1^b^ | 5.59±0.19^a^ | 5.61±0.15^a^ |
| Isoamyl alcohol | 0.63±0.02^b^ | 0.86±0.04^a^ | 0.90±0.06^a^ |
| 2- ethylhexanol | 87.51±1.62^b^ | 92.18±1.25^a^ | 92.65±0.81^a^ |
| 2- phenyl -2- propanol | 5.04±0.11^b^ | 6.15±0.11^a^ | 6.29±0.11^a^ |
| Trans -2-sunflower enol | 0.93±0.03^b^ | 1.11±0.06^a^ | 1.16±0.05^a^ |
| Cis-menthyl-2,8-dienol | 0.34±0.03^b^ | 0.55±0.10^a^ | 0.56±0.06^a^ |
| 2- ethyl -2- propyl hexanol | 2.96±0.11^b^ | 3.18±0.11^a^ | 3.22±0.08^a^ |
| 2- octyldodecanol | 206.92±4.37^b^ | 239.68±6.79^a^ | 248.07±4.8^a^ |
| (Z)-2- octene -1- ol | 13.18±0.51 | 13.28±0.35 | 13.36±0.44 |
| 2- cetyl alcohol | 1.35±0.07 | 1.32±0.09 | 1.33±0.08 |
| Ketones, ng/g |  |  |  |
| 3- hydroxy -2- butanone | 2.48±0.13 | 2.51±0.12 | 2.66±0.21 |
| 3- heptanone | 0.23±0.01 | 0.24±0.01 | 0.25±0.01 |
| Cyclohexanone | 0.54±0.03^c^ | 0.60±0.02^b^ | 0.63±0.03^a^ |
| 2- methyl -2- cyclopentenone | 0.87±0.12^b^ | 0.90±0.06^b^ | 1.06±0.05^a^ |
| Sulfur and heterocyclic, ng/g |  |  |  |
| 2-pentylfuran | 214.53±12.98^b^ | 235.53±7.06^a^ | 238.05±13.98^a^ |
| Esters, ng/g |  |  |  |
| Butyl acetate | 8.36±0.16^b^ | 8±0.32^ab^ | 7.77±0.22^a^ |
| Dimethylphthalate | 37.77±1.9^b^ | 44.26±0.83^a^ | 44.75±1.08^a^ |
| Geraniol isovalerate | 2.52±0.29^b^ | 3.13±0.07^a^ | 3.21±0.12^a^ |
| Diisobutyl glutarate | 2.98±0.05 | 3.13±0.42 | 3.37±0.14 |
| Alkane, ng/g |  |  |  |
| Hexane | 12.52±1.23^b^ | 13.42±1.15^a^ | 13.76±0.82^a^ |
| Tetradecane | 41.98±0.35^b^ | 43±1.23^b^ | 45.72±0.77^a^ |
| 2,6,10 trimethyl-tetradecane | 24.64±1.03 | 25.67±0.85 | 26.63±2.71 |
| Hendecane | 11.67±0.56 | 11.66±0.55 | 11.78±0.48 |

Values are means of 6 replicates per treatment. No identical letters indicate significant differences (*P* < 0.05).
